# Localized coevolution between microbial predator and prey alters community-wide gene expression and ecosystem function

**DOI:** 10.1038/s41396-023-01361-9

**Published:** 2023-01-19

**Authors:** Shane L. Hogle, Liisa Ruusulehto, Johannes Cairns, Jenni Hultman, Teppo Hiltunen

**Affiliations:** 1 Department of Biology, University of Turku, Turku, Finland; 2 Department of Microbiology, University of Helsinki, Helsinki, Finland; 3 Department of Computer Science, University of Helsinki, Helsinki, Finland; 4 Organismal and Evolutionary Biology Research Programme, University of Helsinki, Helsinki, Finland; 5 Natural Resources Institute Finland, Helsinki, Finland

**Keywords:** Microbial ecology, Evolution, Microbial ecology

## Abstract

Closely interacting microbial species pairs (e.g., predator and prey) can become coadapted via reciprocal natural selection. A fundamental challenge in evolutionary ecology is to untangle how coevolution in small species groups affects and is affected by biotic interactions in diverse communities. We conducted an experiment with a synthetic 30-species bacterial community where we experimentally manipulated the coevolutionary history of a ciliate predator and one bacterial prey species from the community. Altering the coevolutionary history of the focal prey species had little effect on community structure or carrying capacity in the presence or absence of the coevolved predator. However, community metabolic potential (represented by per-cell ATP concentration) was significantly higher in the presence of both the coevolved focal predator and prey. This ecosystem-level response was mirrored by community-wide transcriptional shifts that resulted in the differential regulation of nutrient acquisition and surface colonization pathways across multiple bacterial species. Our findings show that the disruption of localized coevolution between species pairs can reverberate through community-wide transcriptional networks even while community composition remains largely unchanged. We propose that these altered expression patterns may signal forthcoming evolutionary and ecological change.

## Introduction

Coevolution is the reciprocal selection imposed by pairwise or multi-way ecological interactions between species [1]. The coevolutionary process is a major force producing phenotypic and genetic diversity both at the microevolutionary scale [2, 3] and across the wider tree of life [4, 5]. Much about coevolution has been learned from studying microbes whose large population sizes, fast generation times, and relatively high mutation rates allow scientists to observe evolution in real-time as it unfolds [6]. Many microbial phenotypes (e.g., antimicrobial production or parasite resistance) have been shaped by antagonistic coevolution whereby hosts/prey evolve resistance to their parasites/predators [7].

Antagonistic coevolution between microbial predators and prey likely affects many ecological and evolutionary processes, including population dynamics [8], the maintenance of local genetic diversity [9, 10], and the enrichment of biodiversity across spatially heterogeneous landscapes [11]. Coevolution between microbial grazers and prey promotes phenotypic and genotypic diversity in marine bacterial populations by selecting for altered cell size, shape, lifestyle, or physiochemical properties of the cell surface, which can increase bacterial survival [9]. For example, phagotrophic protistan grazers have a critical role in controlling the standing stock of bacterial populations [12, 13] and are a significant link in the transfer of dissolved organic carbon from heterotrophic bacteria to higher trophic levels in many microbial ecosystems [14].

Because antagonistic coevolution can drive reciprocal phenotypic diversification of prey/hosts and predators/parasites, this may be a critical process generating intraspecific diversity and driving community composition [15–17]. Intraspecific diversity in a focal species has been shown to alter microbial community composition to a comparable extent to removing a predator [18] or the focal species itself [19]. Phenotypic traits shaped through coevolution may also have pleiotropic consequences for ecological functions unrelated to predator/parasite sensitivity. For example, coevolution between a marine flavobacterium species and two viruses altered the suite of carbon compounds used by the bacterium [20], while rapid resistance evolution in a marine cyanobacterial host has been shown to reduce the effect of viral lysis on dissolved nutrient recycling community stoichiometry [21]. These studies demonstrate that localized coevolution between species pairs can influence microbial community function and composition. However, key questions about the consequences of localized coevolution for ecosystem function and composition remain. Here ask how the coevolution of trophic antagonism between a microbial predator and prey species affects a larger web of interacting microbial species.

**Fig. 1:**
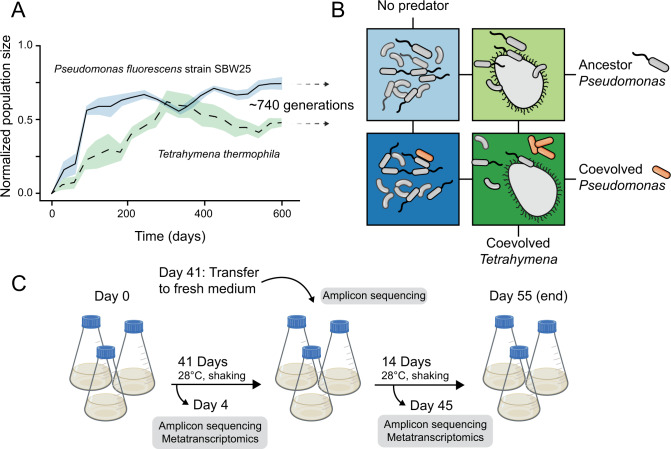
Experimental microcosm overview. **A** Coevolution of *Pseudomonas fluorescens* SBW25 and *Tetrahymena thermophila* in long-term selection lines (mean ± s.e.m. normalized to 0–1, data are from a prior publication [25]). Coevolved ciliate and bacteria were isolated after ~740 generations for use in the main experiment. **B** Treatment scheme of the main experiment. Each box represents the 30 clonal species bacterial community where colors are treatments: bacterial community + ancestor SBW25 without predation (light blue), bacterial community + coevolved SBW25 without predation (dark blue), bacterial community + ancestor SBW25 with coevolved Tetrahymena (light green), bacterial community + coevolved SBW25 with coevolved *Tetrahymena* (dark green). **C** Sampling protocol. Each treatment from **B** was performed in triplicate in 100 ml growth medium. On day 41, fresh media was added to the microcosm. DNA and RNA samples were collected on days 4 and 45. The experiment was terminated after 55 days.

We experimentally manipulated the coevolutionary history of a focal bacterial prey species and ciliate predator in the presence of a bacterial community while measuring cell densities, metabolic potential (per-cell ATP concentration), community composition, and community gene expression (Fig. 1). Based on past studies showing a significant effect of local adaptation on community composition [18, 19], we predicted that altering the coevolutionary history of the predator/prey focal pair would drive overall community composition with largely independent and strain-specific transcriptional responses. Instead, we found that the coevolutionary mismatch between the focal predator and prey species had little impact on community composition but a substantial effect on community-wide transcriptional networks and ecosystem metabolic potential. We conclude by discussing how changes in transcriptional networks may be relevant for eco-evolutionary processes in general.

## Materials and methods

### Experiment overview

Our experiment used a synthetic microbial community of 30 bacterial species and one generalist ciliate predator, *Tetrahymena thermophila*, which successfully preys on each bacterial species. The bacterial species were isolated from soil, aquatic, plant, animal, and human sources, as described earlier [22]. The ciliate *Tetrahymena thermophila* strain 1630/1U (CCAP) was obtained from the Culture Collection of Algae and Protozoa at the Scottish Marine Institute (Oban, Scotland, United Kingdom). The main experiment used a 2 × 2 factorial design which included either a coevolved or ancestor focal prey species, *Pseudomonas fluorescens* SBW25, and included or excluded coevolved ciliates (Fig. 1B). Due to logistical constraints, the main experiment did not include treatment with ancestral ciliates and cannot be used to determine the main or interaction effects of the ancestral ciliate on the bacterial community. Thus, the conclusions here are valid for comparing the effect of the coevolved predator on the prey community to predator-free conditions. Future work could constrain the role of predator coevolution by including an ancestral ciliate treatment. The coevolved predator and prey starting populations were harvested from three replicate coevolution lines after 2 years of coculture (Fig. 1A), made axenic, and then used to start the main experiment. Genomic DNA from the SBW25 clonal ancestor and evolved populations was also sequenced to identify parallel mutations occurring across multiple replicates. Each treatment in the main experiment consisted of three 100 ml replicates starting from either the three coevolved SBW25 replicates or three ancestor SBW25 clones (Fig. 1C). Fresh growth medium was replenished only on day 41, and the entire experiment lasted 55 days. Predator and prey densities and community metabolic potential (cell averaged ATP concentrations) were measured roughly every 4 days, and community composition and gene expression were assessed on days 4 and 45. Per capita ATP concentration is commonly used to indicate ecosystem-level function in microbial communities [23, 24].

### Focal species coevolution

As starting material, the experimental treatments used coevolved ciliates and *Pseudomonas fluorescens* SBW25 from long-term selection lines described earlier [25]. These selection lines were initiated from 10,000 cells ml^−1^ isogenic ciliate and a 48-h culture of clonal SBW25. Cells were incubated in 6 ml of 5% King’s Broth with gentle shaking for 7 days, after which the culture was serially passaged into fresh medium at a dilution of 1:100 (vol/vol), resulting in ~6.6 generations of predator per passage. At week 111 (~740 predator generations) both bacteria and ciliates were isolated from three biological replicates of the SBW25/*Tetrahymena* selection lines following a previously described protocol [25]. Briefly, a one ml aliquot of sample was mixed to a final concentration of 25% (vol/vol) glycerol and frozen at −20 °C. Samples were then thawed (*Tetrahymena* does not survive −20 °C freeze-thaw cycles) and transferred to Proteose Peptone Yeast (PPY) medium. The absence of viable ciliates was verified with microscopy after 24 h of growth. Coevolved ciliates were made axenic with antibiotics (Supplementary Material), and axenicity was checked by plating an aliquot and checking for bacterial growth after a week of 28 °C growth on 50% PPY Extract agar plates. The three replicates of separately purified ciliate and SBW25 were stored at −80 °C until the main experiment.

### Culture conditions, experimental preparations, and sampling

For the main experiment, Ciliates were revived from −80 °C by growing in 50 ml PPY medium for 7 days at 28 °C, after which the cultures were rinsed with M9 salt solution, centrifuged (1700 rcf, 8 min, +4 °C), then resuspended in 25 ml M9 salts. Ciliate cells were counted by microscopy. All bacterial species were added directly to the experiment from thawed −80 °C glycerol stocks, where the cell density of each species was known from prior colony counts. The main experiment consisted of a 2 × 2 factorial design with coevolved or ancestor SBW25 and coevolved ciliate or no ciliate (Fig. 1B). Each condition also included the other 29 clonal bacterial species and was conducted in three replicates (Fig. 1C). Treatments were conducted in 250 ml glass Erlenmeyer flasks with 100 ml of minimal growth medium consisting of 0.2 g l^−^^1^ Reasoner’s 2A broth and 0.1 g l^−1^ cereal grass medium, and 11.5 g l^−1^ 5x M9 minimal salts. The growth medium was prepared by autoclaving and filtering through a 5-µm filter to remove particulate matter. SBW25 was inoculated at 10^7^ cells ml^−1^, while the remaining 29 bacterial species were each inoculated at 10^6^ cells ml^−1^. Ciliates were inoculated at 10^4^ cells ml^−1^, and the same volume of ciliate filtrate (0.45-μm syringe filter) was added to predator-free treatments. Flasks for each treatment were kept at 28 °C with shaking (70 RPM). In total, 10 ml of culture volume was sampled without replacement on days 2, 4, 8, 12, and 16. Additional 10 ml samples were collected on days 23 and 30, but the volume removed from each flask was replaced with M9 salt solution (11.3 g l^−1^) to maintain a constant total volume of 50 ml. On day 41, one 10 ml aliquot was collected, while another 10 ml aliquot was inoculated into 40 ml of fresh growth medium. In total, 10 ml aliquots were collected from the fresh media on days 43, 45, and 53.

### Measurements of consumer and prey densities and community ATP concentration

Bacterial density, predator density, and ATP concentration were measured from 10 ml sample aliquots. Bacterial density was estimated from 1 ml of sample using a spectrophotometer at 600 nm wavelength (OD600). Predator density was measured using 100 µl of fixed sample (10% Lugol’s) in a 96-well plate. Wells were imaged using light microscopy at ×40 magnification with a mounted digital camera and cellSens v1.7 software. Ciliate density was determined from captured images using ImageJ v1.50. The BacTiter-Glo Microbial Cell Viability Assay (Promega) was used to measure ATP concentration with a VICTOR Multilabel plate reader (PerkinElmer) following the manufacturer’s instructions. First, ciliates were removed from samples using a 5-µm filter. Then three technical replicates (50 μl) from each experimental sample were assayed using 12 readings, after which luminescence peaks were averaged across readings and replicates. This mean luminescence intensity was divided by the estimated bacterial density (OD600) in the sample to obtain normalized community ATP concentrations. After measuring prey, predator, and ATP concentrations, one ml of sample was mixed with 0.5 ml of 85% glycerol and archived at −80 °C. Bacterial density was later determined from a subset of the frozen samples using colony counts on 50% PPY agar plates. A separate sample from each time point was collected in an RNAse-free Eppendorf tube and flash-frozen in liquid nitrogen. The samples for RNA extraction were stored at −80 °C.

### Sequencing and bioinformatics

Community DNA was extracted from 0.5 ml of freeze-stored experimental samples using the DNeasy 96 Blood & Tissue Kit (Qiagen). The extraction protocol is described in detail in an earlier publication [16]. Total community RNA was extracted from samples at days 4 and 45 using the Monarch Total RNA Miniprep Kit (New England Biolabs, T2010S). Total RNA was then reverse transcribed to cDNA and sequencing libraries prepared with NEBNext Ultra RNA Library prep Kit for Illumina (New England Biolabs, E7530L). No rRNA depletion was performed on cDNA samples. The V3 and V4 regions of the 16S rRNA gene were amplified from total community DNA as previously described [16]. 16S rRNA gene amplicon libraries were sequenced using a MiSeq System (Illumina) with 300 + 300 bp paired-end reads and V3 chemistry. Genomic DNA from ancestor clones and evolved populations of SBW25 were multiplexed with Nextera Flex DNA barcodes and adapters (Illumina). All cDNA and genomic DNA libraries were sequenced with 150 + 150 bp paired-end reads using a NovaSeq System with S4 flow cell (Illumina).

The 16S rRNA gene amplicon data were quality controlled, mapped, and quantified as previously described [16]. Whole genome sequencing and cDNA read pairs were quality-controlled using bbduk.sh (version 38.61b) to remove contaminants, trim adapters, and right quality trim on base quality (https://sourceforge.net/projects/bbmap/). For mutation calls in resequenced *P. fluorescens* SBW25, reads were mapped to the SBW25 genome (NCBI RefSeq ID: GCF_000009225.2) using BWA-mem v0.7.17 (https://github.com/lh3/bwa) and variants called using Octopus v.0.7.4 [26] in “individual” mode for clonal ancestor samples and “polyclone” mode for coevolved populations using otherwise default parameters. Variants fixed in both the ancestor and coevolved populations were excluded. SnpEff was used to annotate the functional consequences of variants [27]. All cDNA reads were competitively mapped against a combined database of the 30 study species (see Table S1 for RefSeq identifiers) and the *Tetrahymena thermophila* SB210 macronuclear genome (NCBI RefSeq ID: GCF_000189635.1) and partitioned by source genome using bbsplit.sh (https://sourceforge.net/projects/bbmap/). Reads mapping ambiguously to multiple genomes were excluded, and counts were estimated using featureCounts v1.6.2 [28]. Genes from the study species were functionally annotated using EggNOG-mapper 2.0.0 [29], EggNOG 5.0 [30], and antiSMASH v5.0 [31].

### Statistics and data analysis

Nucleotide positions and genes that had undergone significant parallel evolution across independent evolution lines were identified using a previously published statistical framework [32] with nucleotide and gene multiplicity as quantitative measures of evolutionary parallelism. Briefly, nucleotide multiplicity was calculated as the number of replicate populations with a mutation at a given nucleotide site. This was compared to a basic null model assuming a uniform distribution of mutations across all sites in the genome. Gene multiplicity was defined as the number of mutations in gene *i* across all independent replicates multiplied by the ratio of the mean gene length to the length of gene *i*. A null model assuming uniform length-normalized mutational abundance in each gene was specified, and the degree to which log-likelihood of the observed distribution exceeded this null expectation was quantified. To determine the subset of genes enriched in mutations, *p* values for individual likelihood ratio tests were calculated for each gene in the SBW25 genome. The observed *p* values were compared to *p* values calculated under a null distribution, and a critical *p* value was defined such that the FDR was less than 0.05. Genes with a *p* value less than the critical value were deemed to have undergone parallel evolution. See the supplementary material for details.

Ciliate density (cells ml^−1^), bacterial cell density (colony forming units ml^−1^), and normalized ATP concentrations were transformed to the log scale and modeled with hierarchical additive Gaussian process regression implemented in lpgr v1.04 [33] (Supplementary Material). Linear mixed models (estimated using REML and nloptwrap optimizer) were used to predict normalized ATP concentrations and OD_600_ from treatment and day (formula: *y* ~ treatment * poly(day, 2)). The models included treatment, id and poly(day, 2) as uncorrelated random effects (formula: ~1 + treatment | id, ~0 + poly(day, 2) | id). The Shannon index of the bacterial community diversity was estimated from 16S rRNA gene amplicon abundances using DivNet [34]. Significant changes in the Shannon index were detected using the betta method [35]. Between-sample diversity (i.e., Beta diversity) was estimated using Bray–Curtis dissimilarity on relative abundances of 16S rRNA gene amplicon counts and non-metric multidimensional scaling (NMDS) for ordination. Multivariate geometric partitioning in Bray–Curtis dissimilarity space was examined using permutational multivariate ANOVA (PERMANOVA) [36] in vegan v2.5.7. The *p* values were obtained through random permutation of experimental treatments through individual taxa. The assumption of homogeneity of multivariate dispersions implicit in PERMANOVA was tested using a permutational test of dispersions (PERMDISP), and dispersion homogeneity did not differ between treatment categories [37]. Bacterial species abundances were modeled from 16S rRNA gene amplicon counts using beta-binomial regression with corncob v0.2.0 [38].

For visualizing community expression data with ordination, count matrices for each genome were transformed using a regularized log transformation [39]. This regularization produces log_2_ fold changes of read counts for each sample over an intercept, and each gene is analyzed independently. To account for potential confounding variation in underlying gene copy number (i.e., due to shifts in species abundance) between samples, we included a customized normalization factor for within-taxon sum scaling and an amplicon-based estimate of the source taxon’s relative abundance as a model covariate [40]. Thus, any variation in gene expression due to the variation in the underlying DNA template is controlled for in our results. We outline the details of this approach in the Supplementary Material. The rlog transformed abundances for each species-resolved transcriptome constituting greater than 0.5% of all mRNA reads per sample were analyzed with DiSTATIS [41]. DiSTATIS is a three-way generalization of metric multidimensional scaling (classical MDS) that takes multiple distance matrices describing the same sample/event and computes a set of factor scores that best describes the shared similarity structure of all distance matrices (called the compromise scores) as well as partial factor scores that represent the degree of divergence of each distance matrix from the compromise space. Unsupervised clustering of compromise scores was performed using k-means clustering, and the optimal number of group partitions was determined with NbClust v3.0 using the “kl” index [42]. PERMANOVA was used with the Euclidean distance of compromise scores cumulatively explaining 99% of variance in the expression data, and dispersions were tested as described above.

Significant changes in gene expression between experimental conditions for each species-resolved transcriptome were detected using negative binomial generalized linear models, data-driven estimates of experiment-wide variance-mean dependence, and effect size shrinkage as implemented in DESeq2 v1.32 [39]. The same custom sum-taxon scaling normalization approach described above was used for differential expression tests. Differential expression was defined as coding sequence transcripts with a false discovery rate (FDR) adjusted *p* value <0.1 and an absolute log fold change >2. Effect sizes (i.e., fold changes) were moderated using the adaptive t prior shrinkage estimator [43]. Functional enrichment of differentially expressed genes was estimated using the hypergeometric test implemented in clusterprofiler v4.0 [44]. Briefly, KEGG orthology [45] numbers of differentially expressed genes were summarized at the level of KEGG pathways. The hypergeometric distribution was used to test whether KEGG pathway terms occurred within differentially expressed gene subpopulations at frequencies greater than would be expected by chance (FDR-adjusted *p* value <0.05).

## Results

### Parallel mutations in coevolved Pseudomonas SBW25 lines

We first examined the extent of genetic diversity in the coevolved focal species *Pseudomonas fluorescens* SBW25. We looked for parallel mutations at the nucleotide level that had emerged independently in the three replicate *Pseudomonas* populations. Targets of parallel evolution are often under strong selection and provide insight into specific functional adaptations [46, 47]. We found five identical mutations (three deletions and two substitutions) at or greater than 20% frequency across all populations. These mutations did not alter protein products and included four intragenic mutations and one synonymous mutation that was fixed in the *flhB* gene, which encodes a flagellar biosynthesis protein. Synonymous mutations and intragenic mutations with non-neutral fitness consequences may be common in bacteria [48], and our results support the notion that “silent” mutations can be adaptive [49]. The amount of nucleotide parallelism that we observed exceeded our expectations under a simple null model (Supplementary Material, Fig. S1A), indicating that these identical mutations in the three replicate evolution lines are unlikely coincidental.

**Fig. 2:**
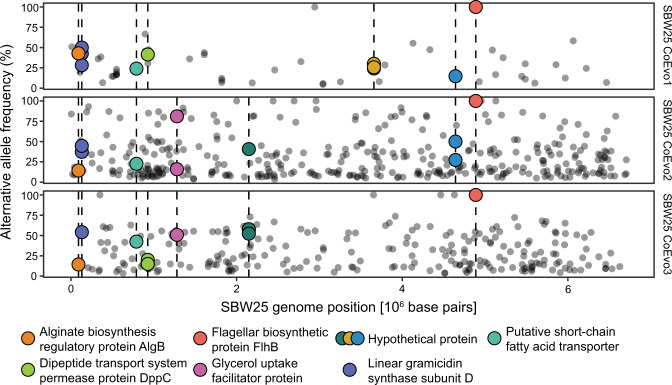
Genomic diversity of coevolved *Pseudomonas* populations from the long-term coevolution experiment. Each row shows mutations called in one of the three independent replicate *Pseudomonas fluorescens* SBW25 populations. The horizontal axis displays the position along the reference SBW25 genome, and the vertical axis displays the frequency of the alternative allele. Points are highlighted if the mutation falls into one of the nine genes evolving in parallel across SBW25 populations (i.e., potential targets of selection). Detailed results are in Table S2 and Fig. S1.

Parallel identical mutations in two or more independent lineages constituted <2.5% of all mutations, so we focused on mutations occurring in the same gene but at different nucleotide positions. We found 53 mutations occurring within the same gene in at least two independent evolution lines (Fig. 2), which was significantly more than expected under neutral evolution (Supplementary Material, Fig. S1B). We next asked which genes were enriched for parallel mutations. We observed nine different genes enriched in mutations across the three parallel evolutionary lines (Fig. 2 and Table S2). These genes, in turn, were functionally annotated with more Two-Component System KEGG terms (KEGG pathway: map02020) than would be expected by chance (hypergeometric test, *p* = 0.03). Specifically, the alginate biosynthesis transcriptional regulatory protein AlgB, the flagellar biosynthetic protein FlhB, and a putative short-chain fatty acid transporter AtoE contained multiple parallel mutations. Two-component signal pathways allow bacteria to sense and respond to external environmental stimuli, and AlgB and FlhB regulate biofilm formation [50] and swimming behavior [51]. Finally, the gene with the highest number of mutations was a nonribosomal peptide synthetase in a putative L-2-amino-4-methoxy-trans-3-butenoic acid biosynthetic gene cluster. This natural product is a small linear peptide toxin and has been shown to inhibit the growth of predatory protists feeding on other *Pseudomonas* species [52].

We previously described coevolved traits in the ciliate populations from the *Tetrahymena*—*Pseudomonas* long-term selection lines, which were used to start the experiments reported here. Coevolved ciliate individuals were larger, faster, and swam in straighter trajectories than isogenic populations that had not been exposed to *Pseudomonas* prey [25]. Ciliate cells with extreme values for these traits forage over a greater search volume [53] and may encounter more prey cells [16]. *Tetrahymena* is nuclear dimorphic and maintains a germline genome and a somatic genome in two separate nuclei. The macronuclear somatic genome consists of 200 different chromosomes, each maintained at a ploidy of 45 during the G1 phase of the cell cycle [54]. This complex genome architecture makes genomic variant calling using short sequencing reads nontrivial. The challenge of resequencing *Tetrahymena thermophila* is beyond the scope of this study, and thus we cannot unambiguously attribute altered predator phenotypes to genotypes here. However, we argue that these phenotypes are most likely caused by heritable mutations to the ciliate somatic genome and not epigenetic mechanisms [25]. First, *Tetrahymena* undergoes phenotypic assortment, where chromosomes are randomly segregated into daughter cells through amitosis. Phenotypic assortment causes the heterozygous somatic genome to rapidly fix adaptive alleles or purge deleterious alleles within only a few generations [55]. Experimental evolution studies have leveraged this phenomenon to demonstrate evolution in *Tetrahymena* over similar timescales as our long-term coevolution experiment [56]. Second, transgenerational epigenetic changes are primarily transmitted through the germline [57], but we grow *Tetrahymena* under conditions that do not permit sexual reproduction. Finally, we purified the coevolved *Tetrahymena* from SBW25 by growing it without bacteria (i.e., selection) for 60 generations before the main experiment (see Supplementary Material). If epigenetic mechanisms caused phenotypic changes in the coevolved *Tetrahymena* lines, we would expect the altered phenotypes to quickly reset to the ancestral form. Instead, we consistently find that coevolved ciliate foraging phenotypes persist in the absence of selection imposed by bacterial prey. Thus, we consider *Tetrahymena* and *Pseudomonas* SBW25 taken from the long-term selection lines to be coevolved.

### Predation drives species density while Pseudomonas coevolution and predation influence community metabolic potential

**Fig. 3:**
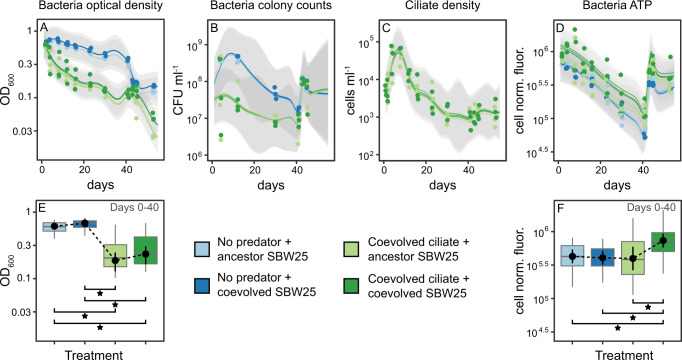
Effect of *Pseudomonas* coevolution on predator and prey density. **A**–**D** Gaussian process regression models for bacterial prey density (OD_600_ and CFU of the whole community), ciliate predator density, and total community metabolic potential (ATP). Lines are the modeled sum of the additive effects with 2x standard deviation (gray bands). Points are experimental observations. **E**, **F** Marginal means and contrasts for linear mixed models predicting OD_600_ and ATP from the treatment category and time with uncorrelated random effects for time and microcosm ID from days 0–40. Marginal means are the average OD_600_ or ATP value at different treatments controlling for other covariates, and contrasts are pairwise differences between marginal means. Statistical significance is marked above the data points (*p* values: *<0.05) The full results from the Gaussian process regression, including variable selection and relevance, are displayed in Fig. S2. The ATP assay fluorescence is normalized to whole community bacterial density. CFU colony forming unit.

We analyzed ciliate and bacterial cell yields and community metabolic potential (represented by optical density-normalized ATP concentrations) during the experiment. Community metabolic potential is regularly used to measure overall ecosystem functioning in microbial communities [23, 24, 58]. We modeled treatment variables through time using Gaussian processes, a Bayesian supervised learning method for solving regression and classification problems. We estimated covariate relevance for the Gaussian processes and selected covariates for final models that explain 95% of the variance with noise [33]. We also used generalized linear mixed models to compare model-derived means of the treatment categories (Fig. 3E, F). Predation had the largest effect on prey density (covariate selected; relevance_OD, CFU_ = 0.36, 0.34), while the coevolutionary history of *Pseudomonas* SBW25 had no effect (covariate not selected; relevance_OD, CFU_ < 5e−4) on predator or prey (Figs. 3A, B and S2). Treatment means were significantly different *(p* < 0.05) between predation but not coevolution categories (Fig. 3E). SBW25 coevolution did not change predator density (Fig. 3C). However, SBW25 coevolution significantly impacted average cellular ATP concentrations but only in the presence of the ciliate (Fig. 3D). Mean per capita ATP concentrations were the same in the no predation and the predation + ancestor SBW25 treatments (*p* > 0.05), but significantly higher (*p* < 0.05) in the predation + SBW25 coevolution treatment (Fig. 3F). Thus, SBW25 coevolution had a significant effect on community-averaged metabolic potential, with the effect contingent upon the coevolved predator.

### Little effect of Pseudomonas coevolution on prey community assembly

**Fig. 4:**
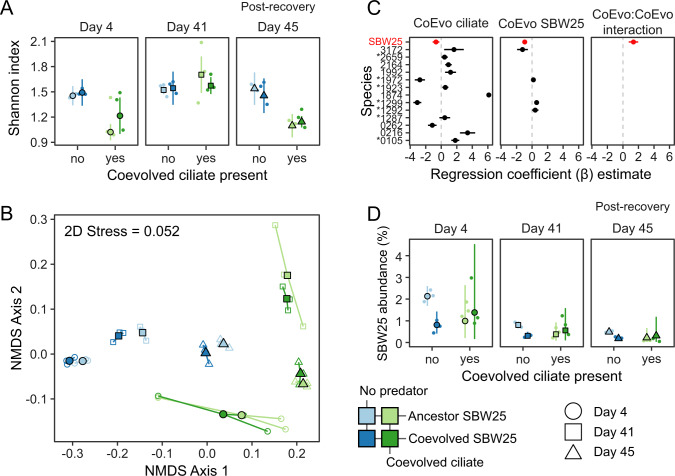
Effects of predation and *Pseudomonas* evolution on bacterial community composition. **A** Shannon index estimates (points and line ranges) and observed values (small points). **B** Two-dimensional non-metric multidimensional scaling (NMDS) of bacterial communities using Bray–Curtis dissimilarity. **C** Estimated beta-binomial regression coefficients. Positive (resp. negative) coefficients indicate a higher (resp. lower) species abundance in the presence of the covariate (panels). Coefficients are only included for species if coefficients have FDR-controlled *p* value ≤0.05. Asterisks denote species that have greater than 1% average relative abundance. **D** Relative abundance *Pseudomonas fluorescens* SBW25 predicted from the beta-binomial statistical model (points and line ranges) and observed relative abundances (small points).

We measured bacterial community composition with 16S rRNA gene amplicon sequencing at the beginning of the experiment (day 4), later before fresh nutrient addition (day 41), and after nutrient addition (day 45). The microcosms were dominated by ten species accounting for over 99% of all amplicon sequences (Fig S3). We used the betta method [35] to model the effect of treatments on the Shannon index estimated with DivNet [34]. Predation reduced the Shannon index of species diversity (Table S3, *β*_predation_ = −0.24, *p* < 1e−5), with the largest reduction on days 4 and 45 (Fig. 4A and Table S3). Both predation (permutational ANOVA: *F* = 73.18, *p* = 0.001) and time (permutational ANOVA: *F* = 26.10, *p* = 0.001) separated experimental communities along the first two axes of an NMDS ordination (Fig. 4B), while the effect of SBW25 coevolutionary history was negligible (Table S4). The effect of SBW25 coevolution on the Shannon index alone or its interaction with predation was negligible compared with predation (Table S3, *β*_SBW25_ = 0.02, *β*_SBW25:pred_ = 0.05, *p* > 0.05).

We used beta-binomial regression to detect differentially abundant taxa across treatment conditions [38]. We considered taxa to be more (resp. less) abundant under a treatment condition if the regression coefficient *β* > 0 (resp. *β* < 0) and *p* < 0.05. Predation significantly altered the abundance of six abundant species (>1% relative abundance, Fig. 4C). SBW25 coevolution slightly increased the abundance of three abundant species (>1%) and decreased the abundance of two rarer species (*Azospirillum brasilense* 3172 and SBW25). Coevolved SBW25 was less abundant than its clonal ancestor in bacterial communities with no predator (Fig. 4D, *β* = −0.89, *p* < 1e−5). In contrast, the mean relative abundance of coevolved SBW25 with the predator was not significantly different from the clonal ancestor (*β* = 0.39 *p* = 0.14), showing that coevolved SBW25 was better able to compete with other bacteria in the presence of a shared predator. SBW25 was the only species with a significant interaction between coevolutionary history and predation covariates in the regression (Fig. 4C), consistent with a growth-defense tradeoff caused by mutations in the coevolved SBW25 lines.

### Response of the active microbial community depends upon both coevolution and predation

We used metatranscriptomic sequencing to investigate the taxonomic composition of the active microbial community during the experimental treatments (Fig. 1B). Taxonomic assignment of RNAs associated with ribosome function (5S, 16S, 23S rRNAs, and tRNAs) and protein-coding RNAs revealed an active community largely similar in composition to the total community inferred from 16S rRNA gene amplicons (Fig. S4). As expected, ribosome-associated RNAs constituted most of the transcriptomes (median = 98%, min = 95%, max = 99%). The focal ciliate species, *Tetrahymena*, recruited 28% of all reads (median = 28%, min = 23%, max = 84%), but recruitment across the genome was uneven, with three unannotated genes recruiting 80% of all *Tetrahymena* transcripts. Nearly all highly expressed ciliate genes were constitutively expressed; thus, we focused our efforts on the activity of the bacterial community.

We next characterized broad patterns in the functional structure of the bacterial community transcriptomes using dimensionality reduction techniques. We selected the 500 most variable coding transcripts in 19 species-resolved transcriptomes (genomes cumulatively recruiting more than 99% of all coding transcripts) on days 4 and 45. We then used these gene expression tables as input to a generalization of principal component analysis [41], allowing us to determine the linear combination of the 19 expression tables that best represented the shared global structure of individual bacterial transcriptomes—i.e., the compromise. The individual bacterial transcriptomes were projected on this compromise space providing a map of individual species’ contributions (Fig. 4).

**Fig. 5:**
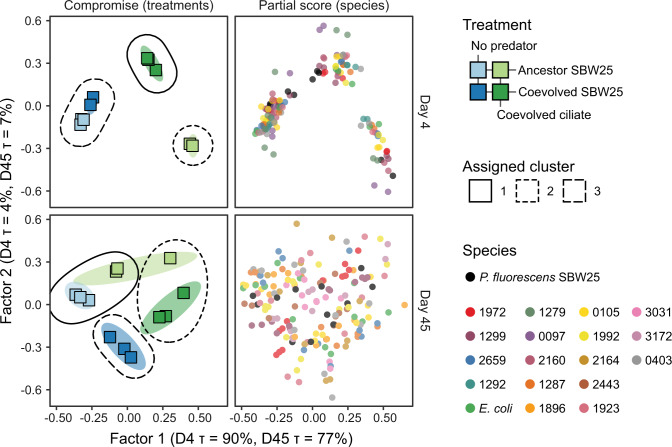
Patterns of community gene expression. DiSTATIS compromise for the 500 most variable coding transcripts in 19 species-resolved transcriptomes (genomes recruiting 95.5% of all coding transcripts) at days 4 and 45. The compromise is a linear combination of the 19 transcriptomes that best represents structure common to the different gene expression matrices. Left—treatment × replicates factor scores (squares). Colored regions are 95% prediction ellipses from 1000 compromise bootstraps. Dashed lines represent clustering results of the compromise (k-means with unsupervised selection of optimal clusters). Three clusters were automatically selected for days 4 and 45. Right—partial factor scores from the 19 transcriptomes projected onto the compromise space. Factor 1 explains 90% and 77% of the variance at days 4 and 45, respectively (*λ*_1_^d4^ = 12.7, *τ*_1_^d4^ = 90%; *λ*_1_^d45^ = 11.6, *τ*_1_^d45^ = 77%). Factor 2 explains 4% and 7% of the variance at days 4 and 45, respectively (*λ*_2_^d4^ = 0.60, *τ*_2_^d4^ = 4%; *λ*_2_^d45^ = 1.10, *τ*_2_^d45^ = 7%).

On day 4, when predator and prey density was at a maximum, the global functional structure of the experimental treatments was clearly separated along two axes, which jointly explained 94% and 84% of the total variance on days 4 and 45, respectively (Fig. 5). The coevolved ciliate drove a community-wide functional response along the first axis analogous to the species shifts observed in the amplicon data. The global effect of *Pseudomonas* SBW25 coevolutionary history was partitioned along axis 2. However, both coevolved and ancestor prey treatments were transcriptionally equivalent without the ciliate predator as supported by unsupervised clustering and permutational ANOVA (Fig. 5 and Table S5). Only in the presence of the coevolved ciliate partner did *Pseudomonas* coevolution drive a clear community functional response along axis 2 (Fig. 5). Predation treatments still aligned mainly with the first axis after nutrient replenishment (day 45), but the relative distances between predation and predator-free treatments were smaller than on day 4. On day 45, prey density rapidly recovered after the nutrient replenishment, while predator density remained low. Like on day 4, *Pseudomonas* SBW25 coevolution at day 45 aligned with the second ordination axis. Unlike day 4, the largest consistent differences between coevolved and ancestor *Pseudomonas* SBW25 treatments were in predator-free treatments. Still, this difference due to SBW25 coevolution at day 45 was small compared to the coevolved *Tetrahymena* and coevolved SBW25 treatments on day 4 (Fig. 5).

### Mechanisms underlying community transcriptomic response to the coevolved focal pair

We next focused on specific genes differentially expressed in the experimental treatments. The overall effect of predation, controlling for SBW25 coevolution, elicited a differential response from 655 genes on day 4 and 1433 genes on day 45 from 17 different bacterial species (Fig. S5). The top 5 most abundant KEGG pathways [45] enriched in these differentially expressed genes were related to amino acid metabolism, ribosomal assembly, ATP biosynthesis, solute transport, and sulfur metabolism. Compared to ciliate predation, the number of differentially regulated genes due to SBW25 coevolution, controlling for the predation effects, was small (*n* = 37 at day 4, *n* = 228 at day 45). There were few functionally enriched KEGG pathways in this gene list, with most transcripts related to ribosome assembly (Fig. S6). We also looked for differentially regulated ciliate genes in response to coevolved SBW25 but identified only 77 *Tetrahymena* transcripts (0.28% of the transcriptome) from day 4, and no transcripts differentially expressed on day 45. *Tetrahymena* downregulated genes from mRNA surveillance and RNA degradation pathways (KEGG pathway: map03015, map03018) in the presence of coevolved *Pseudomonas* (FDR controlled *p* < 0.05). Most ciliate transcripts were assigned to hypothetical proteins without a predicted function.

**Fig. 6:**
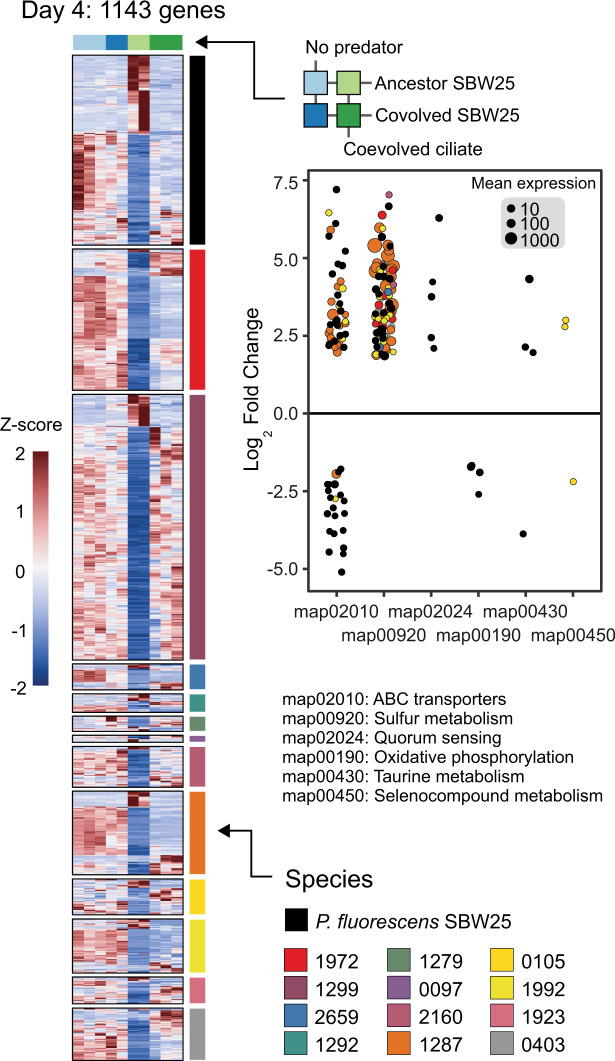
Community response to the coevolved ciliate predator is contingent upon coevolved *Pseudomonas* SBW25. Heatmaps for genes identified as differentially expressed [39] at day 4 (*n* = 1143) in response to *Pseudomonas* coevolution contingent up coevolved ciliate predation while controlling for the overall effects of *Pseudomonas* SBW25 coevolution and coevolved ciliate predation. Each row is a gene colored by species identity and each column is a sample, and columns are colored according to treatment (see Fig. 1 in main text). Heatmap color shows the *Z*-score for each gene across treatment categories. The scatterplot shows the estimated log_2_ fold-change of transcripts (points) from ancestor to coevolved *Pseudomonas* SBW25 treatments contingent upon the presence of the coevolved ciliate (i.e., the light green to dark green column in the heatmap). Genes are only included if they were assigned to KEGG [45] functional pathways that are over-represented in the list of differentially expressed genes from each species using the hypergeometric test. KEGG pathways are shown on the horizontal axis and point size is proportional to the normalized mean expression (Mean expr) level across all treatment conditions.

However, we noticed that the effect of *Pseudomonas* SBW25 coevolution was not consistent across predation treatments. Although few community genes responded consistently to SBW25 coevolution at day 4, many (*n* = 1143 genes, 13 different species) were differentially expressed only in the presence of the coevolved ciliate and coevolved SBW25 (Fig. 6), consistent with the community-wide patterns we observed earlier (Fig. 5). The majority of these differentially abundant genes (*n* = 959) were not detected in either the overall coevolved ciliate or coevolved SBW25 effects from the models (Fig. S7). Unlike day 4, most expression differences on day 45 were attributable to the specific effect of coevolved SBW25 in predator-free treatments (Fig. S7) and were primarily from ribosomal assembly and amino acid metabolism pathways. Comparing expression patterns between days 4 and 45 is complicated by the nutrient spike added on day 41 and the 100-fold reduction in ciliate density on day 45. However, it is clear that the community’s functional response on day 4 was contingent upon the coevolutionary history of both focal species in the experiment.

Community genes differentially expressed in response to the coevolved focal pair were enriched in KEGG functional categories, including ABC transporters (101 genes from three species), sulfur metabolism (89 genes from 7 species), quorum sensing (5 genes from SBW25), ATP biosynthesis/oxidative phosphorylation (4 genes from SBW25), and taurine metabolism (4 genes from SBW25). Sulfur metabolism and transporter genes were equally abundant in both predator-free treatments, expressed at very low levels with the ancestor SBW25 and coevolved ciliate, and then expressed at nearly predator-free levels in the presence of coevolved SBW25 and coevolved ciliate. Genes for the transport and metabolism of organic sulfur and sulfonate-derived compounds (*tauABCD*, *ssuEADCB*) and the assimilatory reduction of sulfate to hydrogen sulfide (*cysJHINCD*) were consistently the most upregulated genes in the presence of the coevolved focal pair (Fig. S8). In addition to their roles in acquiring sulfur [59], these gene clusters have recently been linked to oxidative stress responses in soil bacteria metabolizing hydrocarbons [60], and hydrogen sulfide is a signaling molecule with multifunctional roles protecting against antibiotics and other oxidative stressors [61]. The positive induction of these three gene clusters may be symptomatic of a general stress response unique to the presence of both coevolved focal species. Alternatively, the combined metabolisms of the coevolved pair may have significantly drawn down sulfur compounds to starvation conditions, which induced the expression of genes for scavenging organosulfonates [59]. Thus, sulfur bioavailability, indirectly modulated through coevolved trophic interactions between two focal species, was a key determinant of overall community transcriptional structure.

Some of the most abundant transcripts from *Citrobacter koseri* str. 1287, which dominated in the presence of the predator (Fig. S3), were from type 3 fimbrial genes (*mrkABCDF*) and expressed in the mirror image of the sulfur metabolism and transporters (Fig. S8). This gene cluster was expressed at low levels in both predator-free treatments but at very high levels in the presence of the ancestor SBW25 and the coevolved predator. However, expression returned to predator-free levels in the presence of both the coevolved SBW25 and coevolved ciliate. Type 3 fimbriae are cell surface structures (2–4 nm wide and 0.5–2 μm long) that promote aggregation and are necessary for biofilm formation [62]. This implies that the expression of *Citrobacter* biofilm traits depended upon the coevolutionary history of *Pseudomonas* and *Tetrahymena*. Biofilm production is a niche construction trait that allows bacteria to alter the physical structure of their microenvironment with downstream effects on nutrient diffusion and oxygenation in the vicinity of the biofilm matrix [63]. Thus, the alteration of *Citrobacter* biofilm production due to *Pseudomonas* coevolution had the potential for cascading effects on other species in the community.

## Discussion

Our results demonstrate how the effects of antagonistic coevolution between a focal species pair may cascade through microbial communities by altering multi-species transcriptional networks. We expected that decoupling the coevolutionary history of *Pseudomonas fluorescens* SBW25 from its ciliate predator would perturb bacterial species composition as has been observed before in other microbial communities [18, 19, 64], while RNA sequencing would reveal the functional mechanisms underlying those ecological changes. Instead, we found that the coevolved focal prey species, by itself, had a negligible effect on community structure, which was only significantly altered by the coevolved predator. However, the coevolved prey species had a significant effect on the expression profiles of other bacteria contingent upon the presence of the coevolved predator. This coevolution-dependent effect was also evident in significantly higher community metabolic potential in the microcosms with both coevolved focal species. Later in the experiment, when predator densities dropped considerably, each treatment followed its own ecological and transcriptional trajectory.

Antagonistic coevolution between bacterial hosts and viruses can generate pleiotropic effects like metabolic aberrations, reduced growth, and altered susceptibility to other hosts/viruses when the environmental context changes [65]. Coevolution between bacterivorous protists and bacterial prey likely also generates pleiotropic effects analogous to those between hosts and viruses [9]. However, the impacts of local coevolution and any potential pleiotropic effects on wider community structure and function are not understood. We observed that many mutations from coevolved SBW25 were in genes related to defense traits (e.g., motility, biofilm formation, and small molecule biosynthesis), so we expected these genes to be differentially expressed by coevolved SBW25 in the community experiments. However, none of the SBW25 genes with parallel mutations from the long-term coevolution lines (Fig. 2) were differentially expressed in response to experimental treatments. Indeed, very few genes from any metabolic pathway related to motility or defense phenotypes were differentially expressed in coevolved SBW25. Instead, most differentially regulated genes were involved in nutrient assimilation, sulfur metabolism, and ATP biosynthesis/oxidative phosphorylation. The differentially expressed pathways in the bacterial community were related to sulfur and carbon acquisition with the exception of type 3 fimbriae in *Citrobacter koseri* which contribute to cell adhesion and biofilm formation. Taken together, these patterns show how antagonistic coevolution between one species pair can generate transcriptional diversity across a community of interacting species. This finding represents a link between intraspecific diversity and interspecific phenotypic diversity in microbial ecosystems.

We did not expect organic sulfur metabolism genes (e.g., aliphatic sulfonates) to be differentially regulated in response to SBW25 and *Tetrahymena* coevolution. Inorganic sulfate concentrations in our minimal medium were low (40 μmol l^−1^) relative to commonly used bacteria media (LB; 150 μmol l^−1^ with excess cysteine) and overlapped with sulfate concentrations in natural soils [66]. We speculate that this growth medium was nearly sulfur deficient for the bacterial community and that changes in the coevolved focal pair altered the bioavailability of sulfur in the growth medium, causing downstream effects related to sulfur scavenging [59]. One possibility is that the combined metabolisms of the coevolved ciliate and SBW25 depleted inorganic sulfur concentrations to the extent that it triggered a community-wide sulfur starvation response. Alternatively, the bacterial community may have been responding to aliphatic organosulfonates compounds derived from the *Tetrahymena* predator. For example, Taurolipids, which contain an aliphatic organosulfonate head group, are characteristic lipids of *Tetrahymena* species [67].

This experiment was designed to test how localized coevolution between a focal bacterial species and its ciliate predator affected wider community dynamics and function in a multi-species ecosystem. However, coevolution often occurs within networks of multiple interacting species that vary over time and space [11]. The spatial and temporal heterogeneity of species interactions is an added layer of complexity that needs to be evaluated in subsequent work. However, our findings may be relevant for understanding species’ colonization of new environments. The order and timing of colonization events can affect community assembly and function [68, 69], and our findings add to our understanding of this process by highlighting the potential importance of the coevolutionary history of immigrants. More broadly, our study shows that localized coevolution between a single species pair over ecological time scales can drive functional changes in multi-species transcriptional networks hidden beneath a relatively static community structure. These shifts in community gene expression may be important precursors to the emergence of future adaptive alleles and potentially subsequent species evolution [70]. Thus, our findings also point to gene regulation as a potentially important component of eco-evolutionary change.

## Supplementary information

Supplemental material

## Data Availability

Raw sequencing data are available from the NCBI Sequence Read Archive (SRA) under the BioProject accession number PRJNA818876.

## Code availability

Computer code and data for reproducing all figures and steps in the data analysis are available from https://github.com/slhogle/hambiRNAseq (permanent URL: 10.5281/zenodo.7023198).
